# Selective drainage and rectal misoprostol after laparoscopic myomectomy: a multicenter retrospective study

**DOI:** 10.3389/fmed.2026.1816198

**Published:** 2026-04-15

**Authors:** Qin Jiang, Junqiang Li, Mengling Zhou, Feng Pan, Yong Luo, Fuchuan Jiang

**Affiliations:** 1 Department of Gynecology, Sichuan Provincial People's Hospital, School of Medicine, University of Electronic Science and Technology of China, Chengdu, China; 2 Affiliated Hospital of Southwest Jiaotong University, The Third People's Hospital of Chengdu, Chengdu, China; 3 Chengdu Shuangliu District Maternity and Child Health Care Hospital, Chengdu, China; 4 Affiliated Hospital of Southwest Medical University, Luzhou, China; 5 The First People 's Hospital of Neijiang, Neijiang, China

**Keywords:** laparoscopic myomectomy, minimally invasive gynecology, misoprostol, pelvic drainage, postoperative complications, single-port surgery

## Abstract

**Objective:**

To evaluate the effects of pelvic drainage and postoperative rectal misoprostol on short-term outcomes after laparoscopic myomectomy.

**Design:**

Multicenter retrospective observational cohort study.

**Setting:**

Five tertiary hospitals in Sichuan Province, China, between January 2021 and June 2025.

**Patients:**

Of 302 patients initially identified, 280 met inclusion criteria after exclusions for malignancy, incomplete data, or combined procedures.

**Interventions:**

Patients were categorized into four groups according to surgical approach (multi-port vs. single-port) and postoperative management strategy, allowing evaluation of the independent effects of pelvic drainage and misoprostol within different surgical contexts. The use of pelvic drainage and rectal misoprostol (400 μg) was non-randomized and determined by institutional protocol and surgeon discretion. Perioperative parameters—including operative time, blood loss, drainage characteristics, and postoperative recovery—were extracted from electronic medical records.

**Main outcome measures:**

The primary outcomes were postoperative fever and pelvic infection; secondary outcomes included drainage output and duration, postoperative pain, and length of hospital stay.

**Results:**

Pelvic drainage did not reduce postoperative fever or pelvic infection. In multivariable logistic regression adjusted for relevant covariates (including age, BMI, parity, prior abdominal surgery, myoma size, number of myomas removed, and use of vasopressin or other hemostatic agents), drain placement independently increased the risk of postoperative fever (adjusted OR = 2.30, 95% CI 1.10–4.82, *p* = 0.028). In contrast, postoperative rectal administration of misoprostol significantly decreased the risk of postoperative fever (adjusted OR = 0.53, 95% CI 0.30–0.96, *p* = 0.031) and pelvic infection (adjusted OR = 0.36, 95% CI 0.12–0.97, *p* = 0.043). Among patients with drainage, misoprostol use further reduced total drain volume and duration (both *p* < 0.05). Patients undergoing single-port procedures without drainage also showed low complication rates, with less postoperative pain and shorter hospital stays compared with multi-port cases with drains (all *p* < 0.05).

**Conclusion:**

Pelvic drainage after laparoscopic myomectomy offers no measurable benefit and may heighten postoperative morbidity. In contrast, adjunctive rectal misoprostol effectively lowers the incidence of postoperative fever and pelvic infection.

## Introduction

Uterine leiomyomas are the most common benign tumors of the female reproductive tract, occurring in approximately 30–70% of women of reproductive age (1, 2). For symptomatic patients wishing to preserve fertility, surgical removal remains the mainstay of treatment (3, 4). Laparoscopic myomectomy has largely replaced open surgery due to lower morbidity and faster recovery (5). With further refinement of minimally invasive techniques, single-port laparoscopic myomectomy (SPLM) has emerged as an alternative to conventional multi-port laparoscopy (MPLM), offering comparable outcomes with superior cosmesis and less postoperative pain (6, 7).

The necessity of pelvic drainage after laparoscopic myomectomy remains controversial (8, 9). Although drains are intended to prevent postoperative fluid accumulation and infection (8–10), recent evidence indicates minimal benefit and an increased risk of pain, inflammation, and ascending infection (9–11). Conversely, SPLM—typically performed without drainage—has raised concerns regarding postoperative pelvic fluid retention and febrile morbidity (12, 13).

Misoprostol, a prostaglandin E1 analog, enhances uterine contractility and hemostasis while facilitating absorption of residual pelvic fluid (14). Prior studies have demonstrated its efficacy in reducing blood loss and accelerating recovery after hysteroscopic or laparoscopic procedures (14–16). However, whether misoprostol can compensate for the absence of pelvic drainage in laparoscopic myomectomy, particularly in SPLM, remains unclear.

We therefore conducted a multicenter retrospective cohort study comparing perioperative outcomes among patients undergoing laparoscopic myomectomy with different drainage and misoprostol regimens. Multivariable analyses adjusted for potential confounders were performed to determine the independent effects of drainage and misoprostol on postoperative complications. We hypothesized that pelvic drainage would not reduce postoperative fever or pelvic infection, whereas adjunctive rectal misoprostol would significantly decrease such complications. The primary outcomes were the incidences of postoperative fever and pelvic infection, analyzed separately. Secondary outcomes included drainage characteristics, postoperative pain, and hospital stay.

## Materials and methods

### Study design and population

This retrospective multicenter observational study adhered to the Strengthening the Reporting of Observational Studies in Epidemiology (STROBE) guidelines. No formal sample size calculation was performed because all consecutive eligible patients during the defined study period were included to maximize statistical power. Clinical data were collected from women who underwent laparoscopic myomectomy for benign uterine leiomyomas between January 2021 and June 2025 at five tertiary referral hospitals in Sichuan Province, China. All procedures were performed by board-certified gynecologic surgeons skilled in both multi-port and single-port laparoscopic myomectomy, each with over 50 prior cases, ensuring comparable surgical expertise between groups.

### Patient selection and grouping

Eligible participants were women aged 25–50 years who underwent either MPLM or SPLM with complete perioperative and follow-up records, and histologically confirmed uterine leiomyomas. Exclusion criteria included confirmed malignancy, severe systemic comorbidities, or incomplete essential data. Postmenopausal women were included if they had symptomatic benign fibroids causing compressive symptoms, expressed a desire for uterine preservation, and met all other inclusion criteria. All patients underwent preoperative transvaginal or pelvic ultrasonography, and magnetic resonance imaging when ultrasound findings were inconclusive.

**Figure 1:**
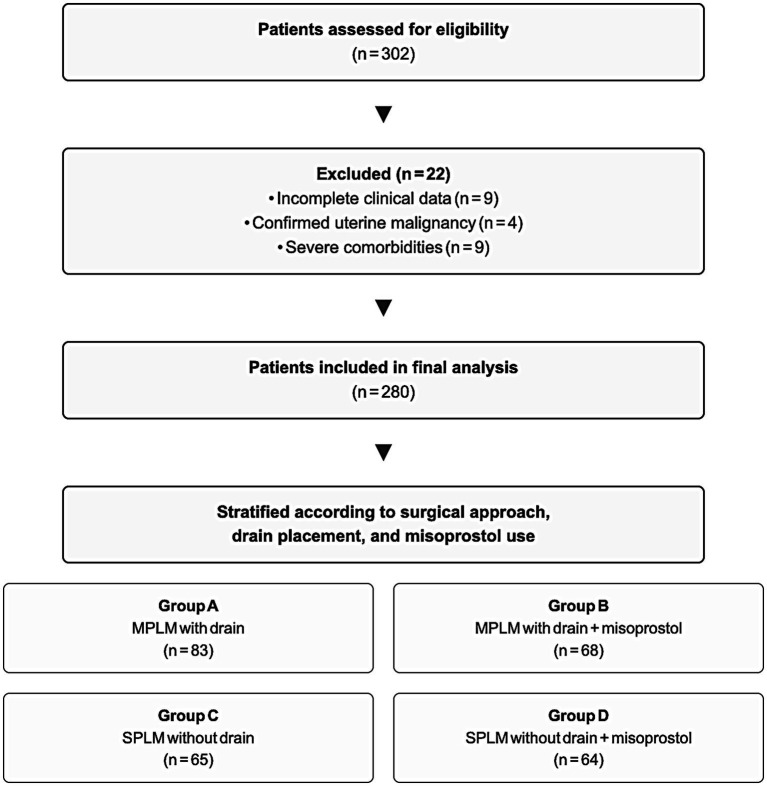
Patient selection and study grouping. Of 302 patients screened, 22 were excluded (incomplete data = 9, uterine malignancy = 4, comorbidities = 9), leaving 280 patients in the final analysis. MPLM, Multi-port laparoscopic myomectomy; SPLM, Single-port laparoscopic myomectomy.

Of 302 patients screened, 22 were excluded (9 for incomplete data, 4 for malignancy, and 9 for severe comorbidities), leaving 280 for final analysis. Patients were classified into four subgroups based on surgical approach, drainage, and postoperative misoprostol use: (A) MPLM with drainage, (B) MPLM with drainage plus misoprostol, (C) SPLM without drainage, and (D) SPLM without drainage plus misoprostol. Patient grouping was non-randomized. Drain and misoprostol use were determined by individual surgeon judgment and institutional practice protocols rather than random allocation. Across centers, SPLM was routinely performed without pelvic drainage, whereas MPLM followed a selective drainage approach based on intraoperative conditions. Consequently, no SPLM-with-drainage and only a few MPLM -without-drainage cases occurred, reflecting institutional tendencies without affecting the analysis. The patient selection process is shown in Figure 1.

Rectal misoprostol (400 μg) was administered once immediately after surgery, with its use and pelvic drainage determined by the surgeon’s discretion according to intraoperative findings and institutional practice. All patients received preoperative prophylactic antibiotics under a unified protocol: a single intravenous dose of 2 g cefazolin within 30 min before skin incision, or clindamycin 600 mg IV for *β*-lactam-allergic patients. Including “center” as a covariate did not alter the results. In patients with drainage, a 6-mm silicone tube was placed in the pelvic cul-de-sac and removed when output fell below 20 mL within 8 h, with daily reassessment if higher. All procedures used standardized laparoscopic techniques across centers.

### Clinical variables

Baseline clinical characteristics included age, body mass index (BMI), parity, postmenopausal status, previous abdominal or pelvic operations, preoperative hemoglobin concentration, and ultrasound-determined size of myomas. Surgical indications such as symptomatic leiomyomas, pelvic pressure, menorrhagia, or infertility were recorded to control for potential confounding in multivariate analyses.

### Surgical technique

Myoma enucleation was performed using sharp and blunt dissection along the pseudocapsule after intracapsular vasopressin injection (1:200 dilution). All visible myomas were sequentially removed while preserving myometrial integrity. Hemostasis was achieved primarily by bipolar coagulation. The uterine defect was closed in two layers with either barbed or conventional 2–0 absorbable sutures, according to surgeon preference. All centers used standardized laparoscopic instruments and bipolar energy for dissection and coagulation.

### Intraoperative and postoperative parameters

Intraoperative parameters extracted from operative notes included operative time, estimated blood loss (EBL), hemoglobin drop, number of myomas and uterine incisions, concomitant procedures (salpingectomy, ovarian cystectomy, or adhesiolysis), use of hemostatic agents, transfusion requirement, and intraoperative complications.

### Postoperative outcomes were categorized as primary or secondary endpoints

The primary outcomes were postoperative fever (≥ 38 °C lasting > 24 h within 7 days after surgery), and pelvic infection defined by persistent fever with elevated inflammatory markers and imaging confirmation of localized infection or abscess. Pelvic infection was diagnosed when persistent fever (> 24 h) was accompanied by elevated inflammatory markers (white blood cell count > 10 × 10^9^/L and/or C-reactive protein > 10 mg/L) and imaging evidence of a localized abscess or fluid collection. Infections were confirmed by positive culture when available; otherwise, cases meeting clinical and imaging criteria were considered presumed infections.

The secondary outcomes included postoperative hemoglobin drop (defined as the difference between preoperative and postoperative hemoglobin levels measured 48 h after surgery), pain score (VAS at 24 h), drainage volume at 24 h (if applicable), time to first flatus (hours), length of hospital stay (days), and any misoprostol-related adverse events, such as transient fever, abdominal cramps, or diarrhea.

### Statistical analysis

Statistical analyses were performed using R software (version 4.3.2). Continuous variables were tested for normality using the Shapiro–Wilk test and expressed as mean ± standard deviation (SD). For normally distributed data, independent-samples t-tests or one-way analysis of variance (ANOVA) were applied as appropriate. Categorical variables were summarized as counts and percentages and compared using the Pearson chi-square (χ^2^) test or Fisher’s exact test when expected frequencies were < 5.

Multivariable binary logistic regression was performed to identify independent predictors of postoperative fever and pelvic infection. Variables with *p* < 0.10 in univariate analysis, as well as clinically relevant factors (age, BMI, parity, prior abdominal surgery, largest myoma size, and surgical approach), were entered into the regression models. Adjusted odds ratios (aORs) and 95% confidence intervals (CIs) were calculated to quantify associations.

Model fit was evaluated using the Hosmer–Lemeshow goodness-of-fit test, where *p* > 0.05 indicated satisfactory calibration. Sensitivity analyses were conducted to verify the stability of the final models. All statistical tests were two-tailed, and a *p* value < 0.05 was considered statistically significant.

### Sample size and power consideration

No formal sample size calculation was performed, as this was a retrospective study that included all consecutive eligible patients during the study period. A post-hoc power analysis based on the observed difference in postoperative fever incidence (8.3% vs. 16.9%) between misoprostol and.

non-misoprostol groups indicated approximately 72% power at *α* = 0.05. This suggests that the current sample size provided adequate, though not optimal, sensitivity for detecting clinically relevant effects.

### Bias control

To minimize bias, all consecutive eligible patients during the study period were included. Data were independently extracted by two investigators and verified by a third researcher to ensure accuracy and reduce information bias. Standardized surgical procedures, anesthesia protocols, and postoperative management were implemented uniformly across institutions to limit performance variability. Missing data were managed using complete-case analysis, and sensitivity analyses were conducted to assess the robustness of regression models and internal consistency of results.

## Results

### Patient characteristics

A total of 280 patients were included: Group A (MPLM with drain, *n* = 83), Group B (MPLM with drain plus misoprostol, *n* = 68), Group C (SPLM without drain, *n* = 65), and Group D (SPLM without drain plus misoprostol, *n* = 64). Baseline demographic and clinical variables, including age, BMI, parity, history of abdominal surgery, postmenopausal status, largest myoma size, and preoperative hemoglobin, surgical indications were comparable among the four groups (all *p* > 0.05; Table 1).

**Table 1:** Comparison of baseline characteristics among four groups.

Variable	Group A (*n* = 83)	Group B (*n* = 68)	Group C (*n* = 65)	Group D (*n* = 64)	Statistic (t/F / χ^2^)	*p-*value
Age (years)	38.54 ± 6.08	37.87 ± 6.72	38.03 ± 6.25	38.63 ± 6.58	*F* = 0.213	0.887
BMI (kg/m^2^)	22.71 ± 3.02	22.93 ± 2.84	22.64 ± 3.21	22.88 ± 2.63	*F* = 0.185	0.908
Nulliparous, n (%)	39 (46.99%)	30 (44.12%)	32 (49.23%)	30 (46.88%)	χ^2^ = 0.423	0.935
Prior abdominal surgery, n (%)	11 (13.25%)	9 (13.24%)	8 (12.31%)	6 (9.38%)	χ^2^ = 0.866	0.834
Postmenopausal status, n (%)	7 (8.43%)	5 (7.35%)	4 (6.15%)	5 (7.81%)	χ^2^ = 0.410	0.937
Largest myoma (cm)	6.22 ± 2.39	6.09 ± 2.32	6.03 ± 2.18	6.01 ± 2.42	*F* = 0.086	0.967
Pre-operative hemoglobin (g/dL)	12.87 ± 1.43	13.06 ± 1.21	13.02 ± 1.29	13.04 ± 1.12	*F* = 0.356	0.784
Primary surgical indication, n (%)	-	-	-	-	χ^2^ = 2.208	0.888
Symptomatic fibroids (pelvic pressure/pain)	46 (55.42%)	35 (51.47%)	34 (52.31%)	33 (51.56%)	-	-
Menorrhagia	27 (32.53%)	24 (35.29%)	20 (30.77%)	21 (32.81%)	-	-
Infertility related to fibroids	10 (12.05%)	9 (13.24%)	11 (16.92%)	10 (15.63%)	-	-

MPLM, multi-port laparoscopic myomectomy; SPLM, single-port laparoscopic myomectomy; Miso, misoprostol. Group A, MPLM with drain; Group B, MPLM with drain + misoprostol; Group C, SPLM without drain; Group D, SPLM without drain + misoprostol. Continuous data: mean ± SD; categorical data: n (%). F values from one-way ANOVA; χ^2^ from Pearson’s chi-square test. All *p* > 0.05 indicate no statistically significant differences in baseline characteristics among groups.

### Intraoperative findings intra- and peri-operative findings

Operative time, EBL, postoperative hemoglobin drop, number of myomas removed, and number of uterine incisions were comparable among the four groups (all *p* > 0.05).

Concomitant procedures were performed in a few cases with no significant inter-group differences (*p* > 0.05). The use of hemostatic agents was also similar among groups (*p* > 0.05). No intraoperative complications or transfusion requirements were reported (Table 2).

**Table 2:** Comparison of intra-and peri-operative parameters among four groups.

Variable	Group A (*n* = 83)	Group B (*n* = 68)	Group C (*n* = 65)	Group D (*n* = 64)	Statistic (F / χ^2^)	*p-*value
Operative time (min)	87.63 ± 24.87	88.09 ± 25.21	91.82 ± 26.77	90.24 ± 25.46	*F* = 0.958	0.412
Estimated blood loss (mL)	126.43 ± 56.12	118.27 ± 51.36	115.36 ± 50.29	118.09 ± 53.44	*F* = 1.035	0.378
Hemoglobin drop (g/dL)	1.02 ± 0.61	0.94 ± 0.49	0.91 ± 0.47	0.84 ± 0.43	*F* = 1.017	0.320
Myomas removed (n)	2.23 ± 0.93	2.05 ± 0.82	2.12 ± 0.97	2.01 ± 0.83	*F* = 0.871	0.457
Uterine incisions (n)	1.41 ± 0.63	1.39 ± 0.60	1.37 ± 0.59	1.33 ± 0.56	*F* = 0.216	0.884
Total concomitant procedures n (%)	7 (8.4%)	6 (8.8%)	5 (7.7%)	6 (9.4%)	χ^2^ = 0.18	0.982
Salpingectomy n (%)	3 (3.6%)	2 (2.9%)	2 (3.1%)	2 (3.1%)	–	–
Ovarian cystectomy n (%)	2 (2.4%)	2 (2.9%)	1 (1.5%)	2 (3.1%)	–	–
Adhesiolysis n (%)	2 (2.4%)	2 (2.9%)	2 (3.1%)	2 (3.1%)	–	–
Hemostatic agents used n (%)	11 (13.3%)	9 (13.2%)	8 (12.3%)	8 (12.5%)	χ^2^ = 0.03	0.998
Transfusion required n (%)	0 (0%)	0 (0%)	0 (0%)	0 (0%)	–	–
Intraoperative complications n (%)	0 (0%)	0 (0%)	0 (0%)	0 (0%)	–	–

Data are expressed as mean ± SD (two decimal places) or n (%). F values derived from one-way ANOVA; χ^2^ for categorical data when applicable. Group A, MPLM with drain; Group B, MPLM with drain+misoprostol; Group C, SPLM without drain; Group D, SPLM without drain+ misoprostol. All *p* > 0.05 indicate no significant inter-group differences.

### Postoperative drainage findings

Among 151 patients who had pelvic drainage (Groups A and B), adjunct use of misoprostol significantly reduced drain volume and duration. The mean drain volume was 61.32 ± 25.47 mL in Group A and 54.83 ± 23.62 mL in Group B (*t* = 2.011, *p* = 0.047); drainage duration was 1.89 ± 0.52 days vs. 1.73 ± 0.43 days (*t* = 2.440, *p* = 0.016; Table 3).

**Table 3:** Effect of misoprostol on drain output among drained patients (Groups A and B, *n* = 151).

Parameter	Group A (*n* = 83)	Group B (*n* = 68)	*t-*value	*p*-value
Total drain volume (mL)	61.32 ± 25.47	54.83 ± 23.62	t = 2.011	0.047 *
Drainage duration (days)	1.89 ± 0.52	1.73 ± 0.43	t = 2.440	0.016 *

Data are expressed as mean ± SD (two decimal places). Statistical test: Independent-sample t test. *p < 0.05 indicates statistical significance. Group definitions are as in Table 1.

### Postoperative recovery and complications

Pain scores at 24 h were highest in Group A (3.52 ± 0.81) and lowest in Group D (2.46 ± 0.72) (*F* = 7.316, *p* < 0.001). Hospital stay was significantly shorter in groups without drain (C and D) (3.41 ± 1.12 and 3.24 ± 0.83 days) than in those with drain (A and B; 4.31 ± 1.47 and 4.13 ± 1.38 days; *F* = 4.704, *p* = 0.004). However, although statistically significant, the mean difference in hospital stay (< 1 day) may not reflect a clinically meaningful improvement. Postoperative fever occurred in 14 (16.87%), 6 (8.82%), 11 (16.92%), and 5 (7.81%) patients in Groups A-D, respectively (*χ^2^* = 10.185, *p* = 0.017). Pelvic infection was observed in 5 (6.02%), 3 (4.41%), 2 (3.08%), and 0 cases (*χ^2^* = 8.023, *p* = 0.046). All infections resolved after antibiotic treatment without reoperation, and no major adverse events were reported. Among nine patients diagnosed with pelvic infection, three had culture-confirmed infection (two *E. coli* and one *Enterococcus faecalis*), while the remaining six met clinical and imaging criteria without culture confirmation (Table 4).

**Table 4:** Comparison of postoperative recovery and complications among four groups.

Variable	Group A (*n* = 83)	Group B (*n* = 68)	Group C (*n* = 65)	Group D (*n* = 64)	Statistic (*F* / *χ^2^*)	*p-*value
Pain score (VAS 24 h)	3.52 ± 0.81	3.19 ± 0.88	2.73 ± 0.79	2.46 ± 0.72	F = 7.316	< 0.001 **
Hospital stay (days)	4.31 ± 1.47	4.13 ± 1.38	3.41 ± 1.12	3.24 ± 0.83	F = 4.704	0.004 **
Postoperative fever n (%)	14 (16.87%)	6 (8.82%)	11 (16.92%)	5 (7.81%)	χ^2^ = 10.185	0.017 *
Pelvic infection n (%)	5 (6.02%)	3 (4.41%)	2 (3.08%)	0 (0.00%)	χ^2^ = 8.023	0.046 *

Data are expressed as mean ± SD (two decimal places) or n (%). F values obtained from one-way ANOVA; χ^2^ from Pearson’s chi-square test. *p < 0.05, **p < 0.01 indicate statistical significance. Group definitions as in Table 1.

Among patients who received misoprostol (Groups B and D), five experienced mild, transient adverse effects: in Group B, two patients developed transient fever, and one had abdominal cramps; in Group D, one patient reported transient fever, and another developed mild diarrhea. All symptoms resolved spontaneously within 24 h without specific treatment. No serious misoprostol-related adverse events were observed (Table 5).

**Table 5:** Summary of misoprostol-related adverse events.

Adverse event	Group B (*n* = 68)	Group D (*n* = 64)	Total (*n* = 132)	Outcome
Transient fever	2 (2.9%)	1 (1.6%)	3 (2.3%)	Resolved spontaneously within 24 h
Abdominal cramps	1 (1.5%)	0	1 (0.8%)	Resolved without treatment
Mild diarrhea	0	1 (1.6%)	1 (0.8%)	Resolved within 24 h
Total	3 (4.4%)	2 (3.1%)	5 (3.8%)	No serious adverse events

### Multivariable logistic regression analysis

Multivariable logistic regression, adjusted for age, BMI, parity, prior abdominal surgery, largest myoma size, number of myomas removed, and use of vasopressin or other hemostatic agents, identified drain placement as an independent risk factor for postoperative fever (adjusted OR = 2.30, 95% CI 1.10–4.82, *p* = 0.028), whereas postoperative rectal administration of misoprostol significantly reduced the risk of postoperative fever (adjusted OR = 0.53, 95% CI 0.30–0.96, *p* = 0.031). Misoprostol use was also associated with a lower risk of pelvic infection (adjusted OR = 0.36, 95% CI 0.12–0.97, *p* = 0.043). None of the adjusted covariates demonstrated a significant association with postoperative complications (Table 6).

**Table 6:** Multivariable logistic regression for postoperative complications.

Outcome	Covariate	Adjusted OR (95% CI)	*p*-value
Fever	Drain present	2.30 (1.10–4.82)	0.028 *
	Misoprostol use	0.53 (0.30–0.96)	0.031 *
Pelvic infection	Drain present	2.66 (0.91–7.80)	0.071
	Misoprostol use	0.36 (0.12–0.97)	0.043 *

Binary logistic regression models were adjusted for all variables listed above. OR, odds ratio; CI, confidence interval. *p < 0.05 indicates statistical significance.

Model calibration was satisfactory according to the Hosmer–Lemeshow goodness-of-fit tests for postoperative fever (*χ^2^* = 6.72, *p* = 0.57) and pelvic infection (*χ^2^* = 5.84, *p* = 0.66). Sensitivity analyses produced consistent findings, confirming the robustness of the regression models.

## Discussion

### Summary of principal findings

This multicenter study evaluated the effects of pelvic drainage and postoperative rectal misoprostol on short-term outcomes following laparoscopic myomectomy. Pelvic drainage did not reduce postoperative fever or pelvic infection. In contrast, adjunctive rectal misoprostol was associated with significantly lower rates of postoperative fever and infection, and among patients with drains, its use further reduced both drainage volume and duration.

Multivariable analysis, adjusted for potential confounders including age, body mass index, parity, prior abdominal surgery, myoma size, and surgical approach, identified pelvic drainage as an independent risk factor for postoperative fever, whereas misoprostol independently decreased the odds of postoperative fever and infection. These findings do not support the presumed benefit of prophylactic pelvic drainage after laparoscopic myomectomy.

### Interpretation and comparison with published literature

Previous large cohort and prospective studies have found no evidence supporting routine pelvic drainage after laparoscopic myomectomy (17–19). Although drains are traditionally used to prevent accumulation of blood and serous fluid, our findings and other evidence indicate that prophylactic drainage provides no proven benefit. Recent reports even suggest that drainage may paradoxically increase postoperative fever and inflammatory responses in minimally invasive gynecologic surgery (8, 20–22). Mechanistically, the drain serves as a foreign body, causing peritoneal irritation, promoting inflammatory exudation, and potentially allowing retrograde bacterial migration (8, 22). Even after adjustment for confounders, drainage remained an independent predictor of postoperative fever, suggesting that its adverse effect is unrelated to surgical difficulty or blood loss. Potential confounding by indication should be recognized, as surgeons tended to use drains in technically demanding or highly vascular cases, which may inherently carry a higher risk of postoperative inflammation.

In this study, MPLM, where drains were more commonly applied, generally involved more trocar sites and wider dissection than SPLM, possibly amplifying postoperative pain and inflammation (23). Conversely, SPLM without drainage was associated with low complication rates and faster recovery, consistent with prior evidence that no-drain laparoscopy is safe and well tolerated (6, 22, 23). These results reaffirm that meticulous hemostasis, adequate irrigation, and minimal surgical trauma can effectively prevent fluid accumulation, rendering routine pelvic drainage unnecessary in most cases (7, 21).

### Mechanistic basis and role of misoprostol

A particularly noteworthy finding is the beneficial role of postoperative rectal misoprostol. A 400 μg rectal dose of misoprostol was selected because this regimen has demonstrated effective prophylaxis against postpartum hemorrhage with fewer adverse effects than higher doses (≥ 600 μg) (24). Misoprostol use significantly reduced postoperative fever and pelvic infection, and among patients with drains, decreased drain output and duration. This effect is biologically plausible: as a prostaglandin E₁ analog, misoprostol enhances uterine contractility, promotes myometrial hemostasis, and facilitates spontaneous clearance of intrauterine and pelvic fluid (14, 15). Improvement in uterine tone and microcirculation likely mitigates local inflammation, thereby lowering febrile morbidity (8). Mild, transient side effects such as low-grade fever or diarrhea were rare and self-limited, consistent with its excellent safety profile. Although misoprostol can transiently elevate body temperature via prostaglandin-mediated thermoregulatory mechanisms, such reactions are short-lived and noninfectious. In this study, brief, self-limiting temperature elevations occurred in a few patients but did not meet the definition of postoperative fever (> 24 h persistence). Overall, both infectious fever and pelvic infection occurred less frequently in misoprostol users.

### Clinical implications

Taken together, the evidence suggests that concerns about postoperative pelvic fluid retention following laparoscopic myomectomy may be overstated. Although the shorter hospital stay in the no-drain groups reached statistical significance, the absolute difference of less than 1 day is unlikely to be clinically meaningful. Pelvic drainage therefore appears unnecessary for most patients when meticulous intraoperative hemostasis and peritoneal fluid evacuation are achieved.

The addition of rectal misoprostol provides a simple, pharmacologic means of improving uterine tone and fluid clearance, likely accounting for its observed benefit in reducing fever and infection. Selective drainage complemented by such pharmacologic intervention therefore represents a safe, feasible, and patient-friendly optimization of postoperative care in minimally invasive gynecologic surgery.

### Strengths and limitations

Strengths of this study include its multicenter design, relatively large sample size, and standardized operative protocols across institutions. The use of multivariable adjustment for surgical approach and other covariates enhanced internal validity and minimized confounding.

However, several limitations should be noted. As this was a retrospective, non-randomized study, selection bias cannot be excluded. Although multivariable regression minimized confounding, residual or unmeasured factors (e.g., surgeon preference, case complexity) may still have influenced outcomes. Propensity score matching was not feasible due to the sample size, but sensitivity analyses confirmed the robustness of our results. Microbiological confirmation was not obtained in all patients with suspected pelvic infection; thus, some diagnoses were based on clinical and imaging criteria alone. This may have slightly overestimated the true infection rate, although the same diagnostic criteria were applied uniformly across all study groups. The small number of febrile and infectious events might have limited statistical power to detect minor differences, although sensitivity analyses showed consistent trends. Pelvic fluid volume was objectively measured only in patients with drains; thus, drainage output served as a surrogate indicator. Inflammatory markers were not routinely monitored, restricting insight into postoperative fever mechanisms. Minor inter-center variations in postoperative management and the fact that all hospitals were located in Sichuan Province may also limit generalizability. A post-hoc power analysis indicated approximately 70–75% power to detect the observed difference in postoperative fever. Despite these constraints, the design reflects real-world practice and provides valuable preliminary evidence to guide larger prospective studies.

## Conclusion

While prophylactic pelvic drainage offers no measurable postoperative benefit, adjunctive rectal misoprostol provides a safe, effective, and mechanistically sound approach to reducing postoperative fever and infection after laparoscopic myomectomy. These findings support a selective drainage strategy supplemented by pharmacologic uterotonic therapy to optimize short-term recovery in minimally invasive gynecologic surgery.

## Data Availability

The raw data supporting the conclusions of this article will be made available by the authors, without undue reservation.
