# Supplementary material for: Selective drainage and rectal misoprostol after laparoscopic myomectomy: a multicenter retrospective study
Source: Front Med (Lausanne). 2026 Apr 15;13:1816198. doi: 10.3389/fmed.2026.1816198 (PMC13126260; doi:10.3389/fmed.2026.1816198)
Supplement: Supplementary file 1 [file Table_1.DOCX]

| **Supplementary Table S1. Multivariable Logistic Regression for Postoperative Complications** | | | |
| --- | --- | --- | --- |
| Outcome | Covariate | Adjusted OR (95% CI) | *P*-value |
| **Fever** | Drain present | 2.30 (1.10 – 4.82) | 0.028 * |
|  | Misoprostol use | 0.53 (0.30 – 0.96) | 0.031 * |
|  | Age (years) | 0.97 (0.92 – 1.02) | 0.270 |
|  | BMI (kg/m²) | 1.04 (0.93 – 1.16) | 0.440 |
|  | Parity (≥ 1 vs 0) | 0.91 (0.48 – 1.74) | 0.781 |
|  | Prior abdominal surgery (yes / no) | 1.12 (0.53 – 2.36) | 0.758 |
|  | Largest myoma size (cm) | 1.03 (0.89 – 1.19) | 0.657 |
|  | Number of myomas removed | 0.95 (0.77 – 1.18) | 0.639 |
|  | Use of vasopressin / hemostatic agents (yes / no) | 1.09 (0.56 – 2.13) | 0.802 |
| **Pelvic infection** | Drain present | 2.66 (0.91 – 7.80) | 0.071 |
|  | Misoprostol use | 0.36 (0.12 – 0.97) | 0.043 * |
|  | Age (years) | 0.98 (0.91 – 1.06) | 0.646 |
|  | BMI (kg/m²) | 1.07 (0.91 – 1.25) | 0.407 |
|  | Parity (≥ 1 vs 0) | 0.88 (0.35 – 2.22) | 0.786 |
|  | Prior abdominal surgery (yes / no) | 1.31 (0.49 – 3.46) | 0.587 |
|  | Largest myoma size (cm) | 1.05 (0.84 – 1.31) | 0.670 |
|  | Number of myomas removed | 1.02 (0.73 – 1.43) | 0.910 |
|  | Use of vasopressin / hemostatic agents (yes / no) | 1.27 (0.49 – 3.27) | 0.621 |
| ****Note:**** Binary logistic regression models were adjusted for all variables listed above. OR = odds ratio; CI = confidence interval. * *P* < 0.05 indicates statistical significance. | | | |
